# MMP‐2 regulates Src activation via repression of the CHK/MATK tumor suppressor in osteosarcoma

**DOI:** 10.1002/cnr2.1946

**Published:** 2023-12-08

**Authors:** Deanna V. Maybee, Christopher R. Cromwell, Basil P. Hubbard, Mohammad A. M. Ali

**Affiliations:** ^1^ Department of Pharmaceutical Sciences SUNY Binghamton University School of Pharmacy and Pharmaceutical Sciences Binghamton New York USA; ^2^ Department of Pharmacology and Toxicology University of Toronto Toronto Ontario Canada

**Keywords:** Csk homologous kinase (CHK/MATK), doxorubicin, matrix metalloproteinase‐2 (MMP‐2), osteosarcoma, Src, Src family kinases (SFK)

## Abstract

**Background:**

Doxorubicin, a first‐line anticancer drug for osteosarcoma treatment, has been the subject of recent research exploring the mechanisms behind its chemoresistance and its ability to enhance cell migration at sublethal concentrations. Matrix metalloproteinase‐2 (MMP‐2), a type IV collagenase and zinc‐dependent endopeptidase, is well‐known for degrading the extracellular matrix and promoting cancer metastasis. Our previous work demonstrated that nuclear MMP‐2 regulates ribosomal RNA transcription via histone clipping, thereby controlling gene expression. Additionally, MMP‐2 activity is regulated by the non‐receptor tyrosine kinase and oncogene, Src, which plays a crucial role in cell adhesion, invasion, and metastasis. Src kinase is primarily regulated by two endogenous inhibitors: C‐terminal Src kinase (Csk) and Csk homologous kinase (CHK/MATK).

**Aim:**

In this study, we reveal that the MMP‐2 gene acts as an upstream regulator of Src kinase activity by suppressing its endogenous inhibitor, CHK/MATK, in osteosarcoma cells.

**Methods and Results:**

We show that enhanced osteosarcoma cell migration which is induced by sublethal concentrations of doxorubicin can be overcome by inactivating the MMP‐2 gene or overexpressing CHK/MATK. Our findings highlight the MMP‐2 gene as a promising additional target for combating cancer cell migration and metastasis. This is due to its role in suppressing on the gene and protein expression of the tumor suppressor CHK/MATK in osteosarcoma.

**Conclusion:**

By targeting the MMP‐2 gene, we can potentially enhance the effectiveness of doxorubicin treatment and reduce chemoresistance in osteosarcoma.

## 1 INTRODUCTION

Matrix metalloproteinases (MMPs) constitute a family of zinc‐dependent endopeptidases overexpressed in several cancer types.^1^, ^2^ In particular, matrix metalloproteinase‐2 (MMP‐2) gained considerable attention for its ability to degrade the extracellular matrix, facilitating detachment from primary tumors and migration to secondary tumor sites, thereby underscoring its critical role in cancer metastasis.^3^ Currently MMP inhibitors employed for cancer treatment predominately target extracellular MMPs, but the lack of specificity (broad targeting of multiple MMPs) renders them less effective in impeding cancer metastasis due to dose‐limiting toxicity.^4^, ^5^ Recent findings reveal that MMP‐2 also localizes to different subcellular compartments, including the nuclei of osteosarcoma U2OS cells. However, the contribution of intracellular MMP‐2 in osteosarcoma cell migration remains largely unexplored.^6^, ^7^

Previous research demonstrated that MMP‐2 expression is regulated downstream of Src activity, a non‐receptor tyrosine kinase and oncogene, through the extracellular signal‐regulated kinases (ERK) pathway.^8^, ^9^, ^10^, ^11^ Src kinase, a member of Src Family Kinases (SFKs), is overexpressed in cancer, and serves a critical role in cell adhesion, invasion and cancer metastasis.^9^ Src's catalytic activity is regulated via phosphorylation at Tyr‐416 for full activation, and Tyr‐527 for inhibition.^12^ Although phosphorylation at Tyr‐527 is indicative of Src inhibition, SFKs' receptor protein‐tyrosine kinases may non‐catalytically bind to the SH2 and SH3 domains to inhibit the Src kinase.^13^, ^14^ The overactivation of SFKs, such as Src, can be attributed in part to the reduced expression of their endogenous inhibitors.^13^

The most common endogenous inhibitors of SFKs include C‐terminal Src kinase (Csk) and the Csk homologous kinase (CHK/MATK).^14^ While Csk is ubiquitously expressed in mammalian cells, CHK/MATK is predominantly found in hematopoietic cells and neurons.^14^, ^15^, ^16^ Csk and CHK/MATK share a similar structural composition with Src, possessing a SH2, SH3 and kinase domain; however, they lack the C‐terminal tail phosphorylation site and N‐terminal myristoyl group,^14^ despite their structural resemblance, the binding domains of Csk and CHK/MATK exhibit differences, as their SH2 domains engage with distinct phosphoproteins and target Csk and CHK/MATK to various cellular compartments.^17^ Both inhibitors were previously reported to catalyze the phosphorylation of the C‐terminal tail tyrosine of Src at Tyr‐527, but recent studies have shown CHK/MATK to be ineffective at phosphorylating Src C‐terminal regulatory Tyr‐527.^13^ Unlike Csk, CHK/MATK has also been shown to directly bind to Src via a non‐catalytic mechanism, thereby preventing autophosphorylation at Tyr‐416 and inhibiting Src activation without affecting Tyr‐527 phosphorylation, and subsequently, inhibit cellular processes such as cell migration.^18^, ^19^

Doxorubicin, an anthracycline antibiotic, is commonly used to treat various cancer types, including osteosarcoma, breast cancer and leukemia.^20^ Specifically, in osteosarcoma, it serves as a first line drug treatment; however, low concentrations result in drug resistance, while at high concentrations cause significant toxicity.^21^, ^22^ Due to doxorubicin's toxic effects on the heart, brain, liver and kidneys, doxorubicin doses need to be lowered in various clinical settings and research has increasingly focused on the cellular mechanisms influenced by different concentrations of doxorubicin.^20^ For instance, a study by Mohammed et al. investigated the impact of a sublethal concentration of doxorubicin on several cancer cell lines, revealing that sublethal concentrations enhances cell migration and invasion through SFK activation in both non‐invasive and invasive cancer cell lines, including U2OS.^23^ Furthermore, doxorubicin has been reported to increase the expression of MMP‐2 and MMP‐9 in cardiac myocytes.^11^, ^24^ These findings align with the study by Mohammed et al., as a sublethal concentration of doxorubicin activates SFKs, augmenting the expression of MMP‐2, and consequently, enhancing cell migration.^23^

The role of intracellular MMP‐2 is increasingly being implicated not only in cancer cell invasion, but also in cell migration.^6^ In our previous studies, we reported that in an osteosarcoma cell line, nuclear MMP‐2 regulates ribosomal RNA transcription through histone clipping, thereby modulating gene expression and cell proliferation.^7^ This discovery has opened up a new avenue of research on the role of intracellular/nuclear MMP‐2 in regulating gene expression, as cleavage of histones will lead to modified chromatin structure and epigenetic alterations regulating gene expression. In the current study, we examined the impact of sublethal concentrations of doxorubicin on enhancing the invasiveness and migration of osteosarcoma U2OS cells in the absence of MMP‐2 gene. We reported that knocking out of MMP‐2 gene considerably hinders osteosarcoma cell migration and inhibits doxorubicin‐induced cell migration. Additionally, we found that the MMP‐2 gene plays a role in regulating Src activation, and consequently, cell migration. We also report that inactivation of MMP‐2 inhibits Src activation through upregulating the endogenous Src inhibitor, CHK/MATK. Lastly, although a sublethal concentration of doxorubicin promotes osteosarcoma cell migration, combining this treatment with CHK/MATK overexpression in osteosarcoma cells hinders, or at least partially attenuates, cell migration. We conclude that a deeper understanding of the role of intracellular/nuclear MMP‐2 in cell migration may pave the way for new strategies to effectively target cancer migration and metastasis.

In summary, this study investigates the role of MMP‐2 in cancer migration and metastasis in osteosarcoma. The study reveals that MMP‐2 has a dual role in cancer progression. It not only degrades the extracellular matrix to facilitate tumor detachment and migration, but it also affects cell migration through intracellular processes such gene expression changes. The study's findings highlight the regulation of Src activation by MMP‐2 and its interaction with the endogenous Src inhibitor CHK/MATK, which presents new possibilities for targeted cancer therapies. Additionally, the study demonstrates that sublethal concentrations of doxorubicin can promote cancer cell migration, but this effect can be countered by inactivating MMP‐2 and/or overexpressing CHK/MATK. This suggests a potential strategy to mitigate doxorubicin‐induced cell migration while still utilizing its anti‐cancer properties. Overall, these findings contribute to our understanding of cancer cell migration and metastasis, offering insights for more effective therapeutic approaches, particularly in osteosarcoma treatment and potentially in other cancers as well, with the aim of improving patient outcomes.

## 2 MATERIALS AND METHODS

### 2.1 Antibodies and reagents

The reagents and antibodies purchased include: Dulbecco's modified Eagle's medium (DMEM), Corning; fetal bovine serum (FBS), Krackler; Src (2109 T), phospho‐Src Family (Tyr416) (6943 T), phospho‐Src (Tyr527) (2107 T), CHK/MATK (20729S), Csk (4980 T), beta‐Actin (4970 T), GAPDH (97 166 T), Cell Signaling Technology; MMP‐2 (ab92536), Abcam; Doxorubicin hydrochloride (D1515‐916), Sigma; Lenti ORF particles, MATK (mGFP‐tagged)‐ Human megakaryocyte‐associated tyrosine kinase (MATK) transcript variant 1, Origene.

### 2.2 Cell culture

Human osteosarcoma cell line (U2OS; ATCC‐HTB‐96) were cultured at 37°C in a humidified 5% CO_2_ atmosphere in DMEM (Krackler, 23‐10‐013‐CM‐PK) supplemented with 10% FBS (Krackler, 23‐35‐010‐CV). U2OS WT+ GFP‐MATK stables were additionally supplemented and cultured with 4 μg/mL puromycin (ThermoFisher, A1113803). MMP‐2 knockout U2OS cells were generated by CRISPR/Cas‐9, as previously described.^7^

### 2.3 Lentiviral transduction

U2OS WT cells were seeded at 300000 cells in a 35 mm dish (VWR, 10861‐656) (50% confluency) and cultured for 24 h under standard culture conditions. The number of viral particles were calculated according to the multiplicity of infection (MOI), 5 MOI per U2OS cell, totaling 1.5 × 10^6^ total transducing units needed. The appropriate number of lentiviral particles (MATK‐GFP‐tagged), culture medium, and 4 μg/mL polybrene (Millipore Sigma, TR‐1003‐G) to the total volume of 500 μL was added to the 35 mm dish and cultured overnight. The next day after transduction, the medium containing lentiviral particles was removed and replaced with fresh medium. 72 h after transduction, a stable cell line was generated using 4 μg/mL of puromycin (ThermoFisher, A1113803) (drug resistant marker).

### 2.4 qPCR

U2OS cells were seeded on 10 cm plates (VWR, 10861‐680) under standard culture conditions and incubated for 24–48 h to reach 100% confluency. Once cells reached confluency (~2.0 × 10^6^ cells) RNA from cells were isolated using RNeasy Plus Mini Kit (74 134, Qiagen). Using the RNA, cDNA was synthesized using qScript cDNA SuperMix and protocol from Quantabio (101414‐102). The qPCR primer cocktails were made using PerfeCTa SYBR Green FastMix (101414‐276), forward primer, reverse primer (Real Time Primers®) and RNase/DNase free H_2_O (Fisher Scientific, AM9914G). The following primers were used as follows: HART (Forward‐5′‐CTGCGATGGTGGCGTTTTTG‐3′, Reverse‐5′‐ACAGCGTGTCAGCAATAACC‐3′), MMP‐2 ((VHPS‐5759) Forward‐5′‐TTGACGGTAAGGACGGACTC‐3′, Reverse‐5′ACTTGCAGTACTCCCCATCG‐3′), CDC2 ((VHPS‐1712) Forward‐5′‐CATGGGGAT TCAGAAATTGA‐3′, Reverse‐5′‐ATTCGT TTG GCTGGA TCATA‐3′), MATK ((VHPS‐5576) Forward‐5′TCATGACGAAGATGCAACAC‐3′, Reverse‐5′CTTGCTCTCCAGGTACTCCA‐3′), CSK ((VHPS‐2263) Forward‐5′‐TTCTGCAAAGGAGAC GTG CT‐3′, Reverse‐5′‐GGTTTAATGAGGCGCGTACA‐3′), and SRC ((VHPS‐8846) Forward‐5′‐GGGTGATGTTTGACCTTCAG‐3′, Reverse‐5′‐TAGGCA CTCTTTTCCCTCCT‐3′). In a 96 well qPCR plate (Biorad, HSP9601), 18 μL of primer cocktail was added and 2 μL of cDNA, accordingly. qPCR was run using BioRad cfx96 and fold gene expression was calculated. The ΔCT was calculated by subtracting the average CT of the housekeeping gene HPRT1 from the average CT of the gene of interest. The ΔΔCT was calculated by subtracting the ΔCT of the sample (WT or KO) by the ΔCT of the WT (control/reference). The fold gene expression equals 2^−(ΔΔCT)^.

### 2.5 Western blotting

Cell lysis samples were run through gel electrophoresis using 10% SDS‐PAGE gels and proteins were electrotransfered onto PVDF membranes (97062‐900, VWR). The membranes were then blocked with 5% dried skimmed milk (Village Farm Instant Nonfat Dry Milk) in TBS‐T (50 mM Tris pH 8.4, 0.9% NaCl, 0.05% Tween‐20) for 1 h and incubated overnight at 4°C in the presence of previously listed primary antibodies in 5% milk. Prior to incubating the membrane in the secondary antibody for 1 h at 25°C, it was washed with TBS‐T 3 times for 10 min. each. The membrane was developed using Radiance Plus Reagents (AC2103) and chemiluminescent bands were revealed (Azure Biosystems).

### 2.6 Gelatin zymography

Cell lysis samples (20 μg of protein per sample) were prepared with 4× Laemmli buffer without 2‐mercaptoethanol (BioRad, 1 610 747) and samples were not boiled. Samples were electrophoresed through 8% tris‐glycine polyacrylamide gel with 0.1% gelatin and gels were washed with 2.5% Triton X‐100 (Millipore Sigma, T8787‐250ML) 3 times for 20 min each. Gels were incubated in an incubation buffer (50 mM Tris‐HCl, pH 7.5, 5 mM NaCl, 1 mM CaCl_2_,) overnight at 37°C. The next day, the gel was stained with 0.05% Coomassie Brilliant Blue G‐250 stain (Sigma B1131) for 1 h and destained with a destaining solution (30% methanol, 10% glacial acetic acid) overnight at 25°C. Bands were visualized (Azure Biosystems, N600).

### 2.7 Cytotoxicity assay

In a 96 well plate (VWR, 10861‐666), 5000 U2OS WT cells were seeded and cultured for 24 h under standard culture conditions. Treated cells with various concentrations of doxorubicin hydrochloride for 24 and 48 h. After 24 and 48 h, LDH activity was measured using reagents from CyQUANT LDH Cytotoxicity Assay Kit (C20300, invitrogen). Absorbance was read at 490 nm and 680 nm to determine LDH activity. Percent cytotoxicity was calculated using the following: [(Compound‐treated LDH activity‐Spontaneous LDH activity)/(Maximum LDH activity‐Spontaneous LDH activity)] × 100.

### 2.8 Transwell migration assay

In 24 well plates with transwell chamber inserts (Costar 3464, Corning), U2OS WT and MMP‐2 KO cells were seeded, separately, in the upper chamber at 20000 cells in 200 μL serum‐free DMEM. In the bottom chamber of the well 800 μL DMEM supplemented with 10% FBS was added. The plates containing triplicates of WT and KO were cultured for 24 h under standard culture conditions. The following day, non‐migrating cells were removed from the upper chamber and migrating cells were fixed with 70% ethanol (VWR, BT143215) and stained with 0.2% crystal violet (Millipore Sigma, V5265‐250ML). Membranes were left to dry and images were taken at 10× using Olympus microscope (IX73 model).

### 2.9 Wound closure assay

In 6 well plates (VWR, 10861‐696), U2OS WT, MMP‐2 KO and GFP‐MATK stable cells were cultured, separately, for 24 h under standard culture conditions to reach 100% confluency (~0.5 × 10^6^ cells). Once confluent, a vertical scratch was formed down the middle of the well using a 200 μL pipette tip (VWR, 76323‐390). Cells were washed with 1× PBS (21‐040‐CMR Corning, and OPTI‐MEM (Thermofisher Scientific, 31 985 070) was added to each well. The appropriate concentrations of doxorubicin were added to the OPTI‐MEM in respective wells. Images of the wound were taken at 0, 24 and 48 h time points. The percent wound closure at 24 and 48 h were calculated as the following: [(Initial wound width‐final wound width)/(initial wound width) × 100].

### 2.10 Statistical analysis

The results of this study are expressed as mean ± standard error of the mean. The statistical significance between the mean values were analyzed by Student's t‐test or one‐way ANOVA and Dunnett's multiple comparisons test using GraphPad Prism 9. Significant differences; *p < .05, **p < .01, ***p < .001, ****p < .0001.

## 3 RESULTS

### 3.1 MMP‐2 knockout impairs cell migration in osteosarcoma

**FIGURE 1:**
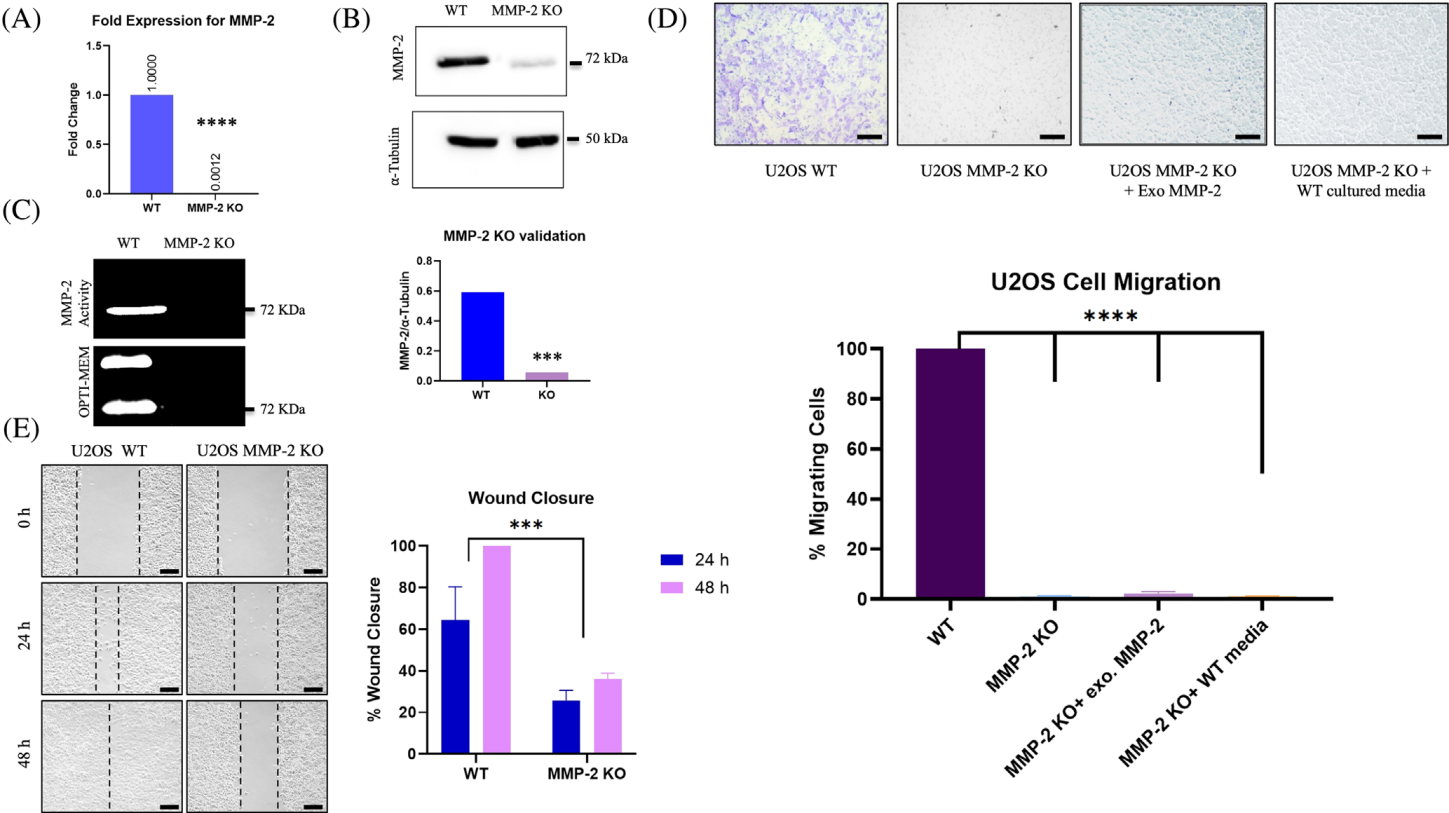
Effect of knocking out MMP‐2 gene on osteosarcoma cell migration. (A). Fold change MMP‐2 gene expression for U2OS WT and MMP‐2 KO cells (*p* < .0001, t‐test). (B). Above; MMP‐2 protein levels for WT and MMP‐2 KO cells. Below; quantification of MMP‐2 protein levels to α‐tubulin in WT and KO cells (*p* < .0001, t‐test). (C). MMP‐2 activity levels in WT and KO cell lysates and excreted MMP‐2 in cultured OPTI‐MEM medium, validating our MMP‐2 KO cells. (D). Above; transwell migration assay of U2OS WT, MMP‐2 KO, MMP‐2 KO cells with exogenous MMP‐2, and MMP‐2 KO cells with WT cultured media containing secreted MMP‐2 at 10× magnification. Black scale bar in right bottom corner of each image represents 200 μm. Below; quantification of percentage cells migrated after 24 h after normalizing to WT (*p* < .0001, one‐way ANOVA). (E) Left; wound closure assays of U2OS WT and MMP‐2 KO (*n* = 3) at 10× magnification. Black scale bar in right bottom corner of each image represents 200 μm. Right; quantification of % wound closure at 24 and 48‐h time‐points (*p* < .001 between WT and KO, one‐way ANOVA). Inactivating the MMP‐2 gene significantly impeded the migration of U2OS cells, and the addition of exogenous or secreted active MMP‐2 did not recover their migratory phenotype.

To validate our U2OS MMP‐2 knockout cell line, we analyzed the mRNA levels and examined the fold change of MMP‐2 gene expression between the wildtype (WT) and MMP‐2 knockout (KO) (Figure 1A). qPCR analysis of MMP‐2 mRNA showed a significant difference between the KO and WT, with the KO displaying an 833‐fold decrease in MMP‐2 transcripts compared to the WT cells (Figure 1A) (*p* < .0001). The MMP‐2 gene inactivation in KO was also confirmed at the protein level by performing western blot (Figure 1B) (*p* < .0001) and analyzing the activity of MMP‐2 in our WT and KO cells by gelatin zymography (Figure 1C).

In order to assess the impact of MMP‐2 KO on cancer cell migration, transwell migration assays were conducted on U2OS WT and KO cell lines. Our findings demonstrated that while the WT cells displayed a high level of migration ability, the MMP‐2 KO cells exhibited complete inhibition of cell migration (Figure 1D) (*p* < .0001). Interestingly, adding active MMP‐2 enzymes exogenously to the serum‐free media of KO cells did not restore cell migration. Likewise, adding the culture media from WT cells, that contains active secreted MMP‐2, to the KO cells did not enhance cell migration. It is noteworthy that although prior research had established that MMP‐2 inhibitors results in inhibition of tumor invasion and angiogenesis attributable to the activity of extracellular MMP‐2,^25^ our study is the first to demonstrate a significant inhibition in osteosarcoma cell migration as a result of inactivating MMP‐2 gene in the presence of active extracellular MMP‐2.

To further examine the effect of MMP‐2 KO on the migratory ability of osteosarcoma cells, we conducted wound closure assays on both the WT and MMP‐2 KO cells for the duration of 48 h until complete wound closure was observed in the WT (Figure 1E). Minimal wound closure (approximately 35% closure vs. 100% closure in WT) was observed in the MMP‐2 KO at 48 h time‐point (Figure 1E) (*p* < .001). Thus, knocking out the MMP‐2 gene in osteosarcoma cells, in the presence of active extracellular MMP‐2, results in a significant inhibition of cancer cell migration, suggesting that the MMP‐2 gene, rather than extracellular protein, plays a major role in cancer cell migration pathways.

### 3.2 Sublethal concentrations of doxorubicin enhance osteosarcoma cell migration in an MMP‐2‐dependent manner

**FIGURE 2:**
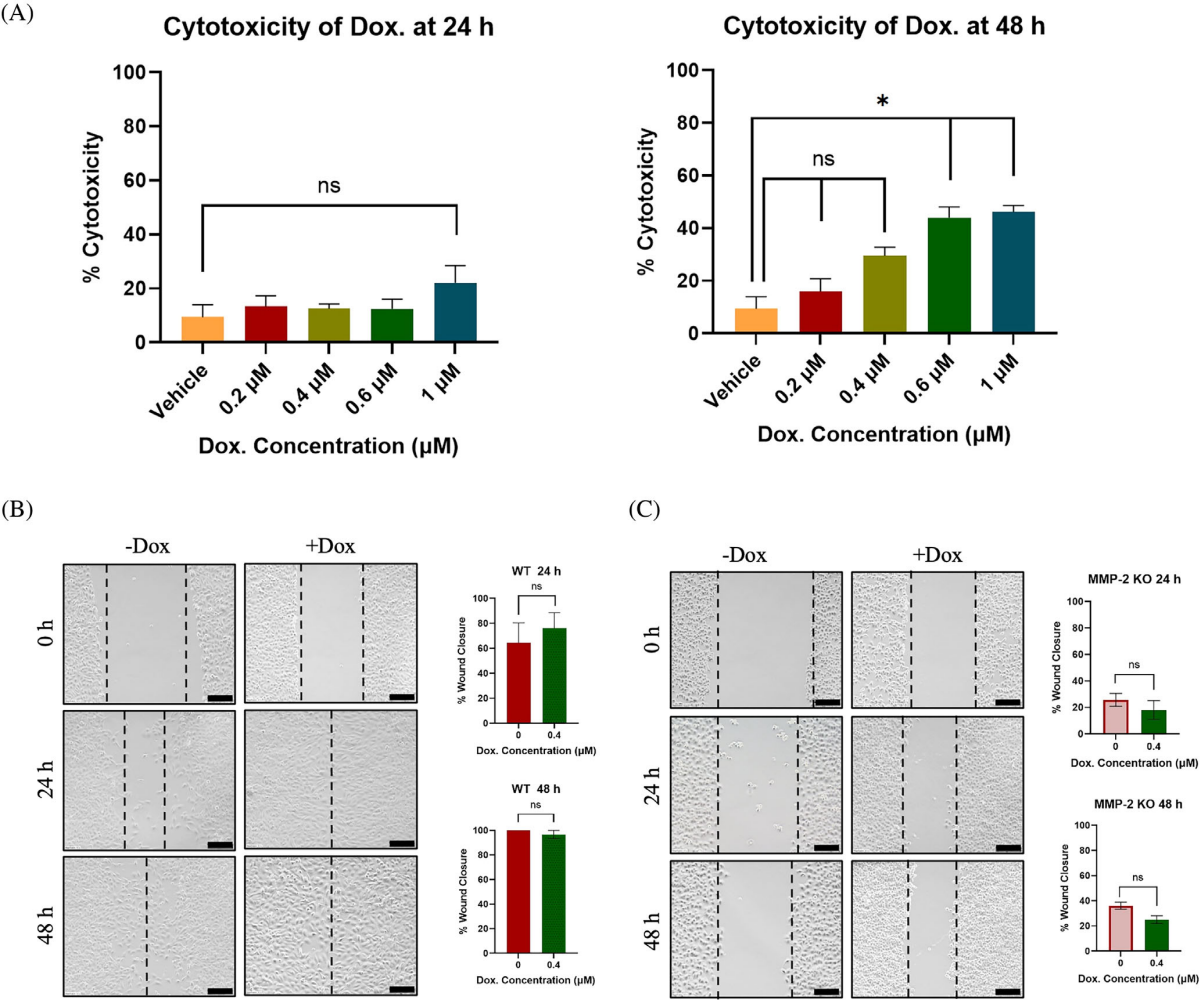
Role of MMP‐2 in enhancement of cell migration by sublethal concentrations of doxorubicin. (A) Left; percent cytotoxicity of doxorubicin at various concentrations after 24 h (0, 0.2, 0.4, 0.6, and 1 μM). Right; percent cytotoxicity of doxorubicin at 48 h, showing 0.4 μM doxorubicin as a sublethal concentration of doxorubicin for U2OS cells, as it is statistically non‐significant from the vehicle (*p* > .05, one‐way ANOVA). (B) Left; wound closure assays for U2OS WT −/+ 0.4 μM doxorubicin at 24 and 48‐h at 10× magnification. Black scale bar in right bottom corner of each image represents 200 μm. Right; quantification of % wound closure at 24 h (upper) and 48 h (lower) time‐points (*n* = 3). (C) Left; wound closure assays for U2OS MMP‐2 KO −/+ 0.4 μM doxorubicin at 24 and 48 h at 10× magnification. Black scale bar in right bottom corner of each image represents 200 μm. Right; quantification of % wound closure at 24 h (upper) and 48 h (lower) time‐points for WT and KO (*n* = 3). Although the sublethal concentration of 0.4 μM doxorubicin enhances U2OS cell migration, this enhancement in cell migration is lost when MMP‐2 gene is inactivated.

Previous research by Mohammed et al. revealed that sublethal concentrations of doxorubicin enhances cell migration in several cancer cell lines, including U2OS.^23^ To determine the concentrations of doxorubicin causing a sublethal effect on our U2OS cell line, a LDH release assay was performed. A sublethal concentration was determined by the percent cytotoxicity having non‐significant difference from the vehicle at the respective time point. The highest sublethal concentration of doxorubicin on U2OS was 0.4 μM for both 24‐ and 48‐h treatments (*p* > .05), whereas the percent cytotoxicity of 0.6 μM and 1 μM doxorubicin were statistically significant from the vehicle at 48 h (*p* < .05) (Figure 2A). This doxorubicin sublethal concentration enhanced cell migration in WT cells using wound closure assays. Although the trend was clear the *p*‐value equals 0.0523 (non‐significant), with nearly complete wound closure (approximately more than 80% closure) observed at 24 h, compared to approximately 60% wound closure in the untreated sample (Figure 2B), supporting a previous study.^23^ Despite these results in the WT, the sublethal concentration of doxorubicin failed to enhance cell migration in MMP‐2 KO cells, which continued to show minimal migration at 24 and 48 h without or with 0.4 μM doxorubicin treatment (25% vs. 20%, respectively) (*p* = .1086 and *p* = .1132, respectively) (Figure 2C).

Our findings indicate that sublethal concentrations of doxorubicin augment cell migration in WT U2OS cells. However, this increase in cell migration is negated when the MMP‐2 gene is knocked out. This impairment in cell migration in MMP‐2 KO cells provides further evidence of the MMP‐2 gene's involvement in cancer cell migratory pathways.

### 3.3 MMP‐2 gene mediates doxorubicin‐induced phosphorylation of Src at Tyr‐416

**FIGURE 3:**
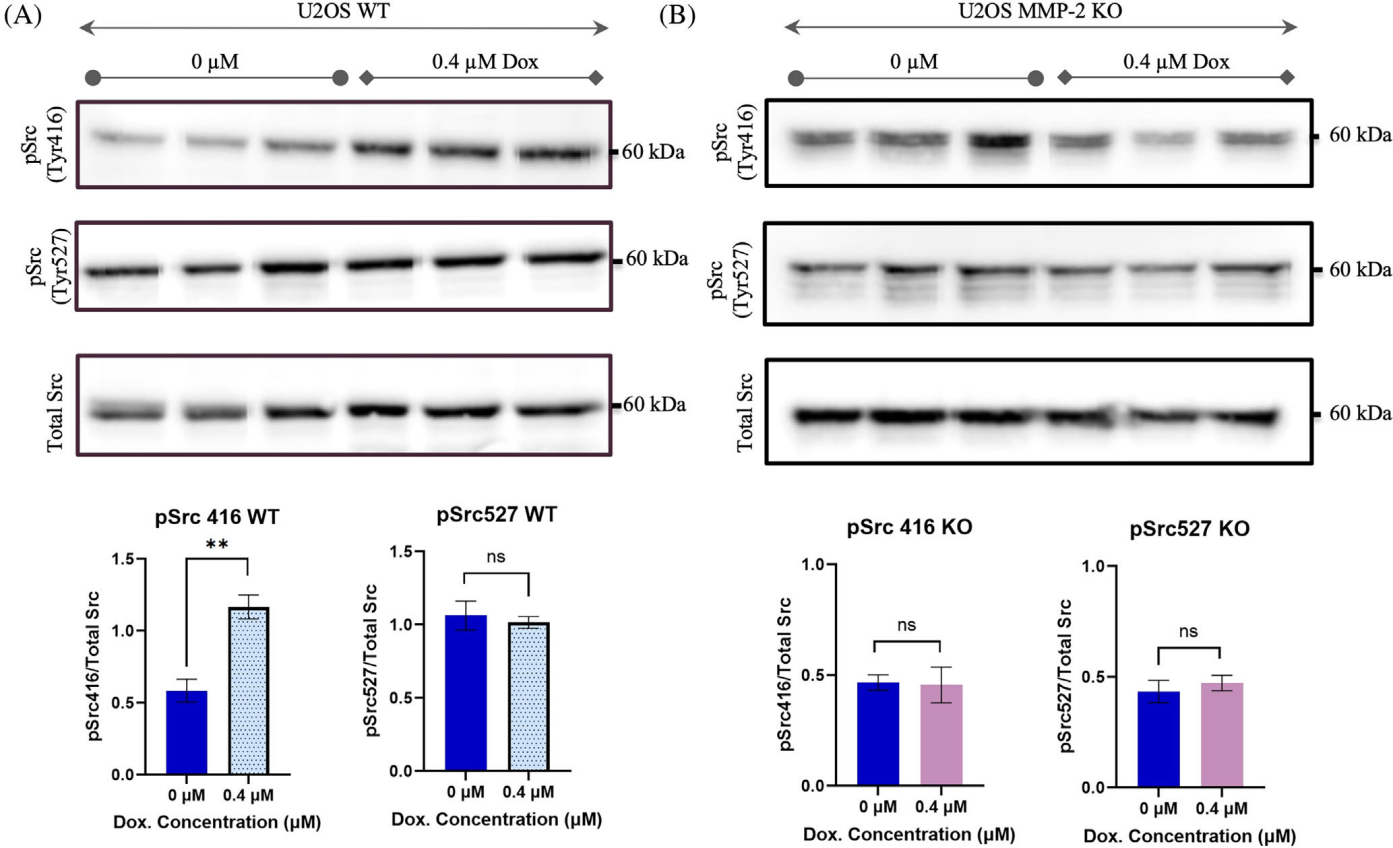
MMP‐2 regulates Src phosphorylation at Tyr‐416. (A). Top; levels of Src phosphorylation at Tyr‐416 and Tyr‐527 after 0 and 0.4 μM treatment at 24 h for U2OS WT (*p* < .01 and *p* > .05, t‐test respectively). Bottom; quantification of pSrc at Tyr‐416 (left) and pSrc at Tyr‐527 (right) for U2OS WT. (B). Top; levels of Src phosphorylation at Tyr‐416 and Tyr‐527 after 0 and 0.4 μM treatment at 24 h for U2OS MMP‐2 KO. Bottom; quantification of pSrc at Tyr‐416 (left) and pSrc at Tyr‐527 (right) for U2OS MMP‐2 KO (*n* = 3) (*p* > .05, t‐test). In the presence of a sublethal concentration of doxorubicin, Src activation and phosphorylation at Tyr‐416 occurs in an MMP‐2‐dependent manner.

Mohammad et al. showed that increases in Src phosphorylation at Tyr‐416, representing active Src, occurs when cancer cells are treated with sublethal concentrations of doxorubicin.^23^ The increase in phosphorylated Src at Tyr‐416 serves as an indicator of Src activation, which plays an important role in cytoskeletal reorganization and cell migration.^12^, ^26^ We sought to investigate Src activation induced by sublethal concentrations of doxorubicin in both WT and MMP‐2‐KO cells. As a result, the WT and MMP‐2 KO cells were treated with 0.4 μM doxorubicin for 24 h and pSrc at Tyr‐416 was measured (Figure 3A). Western blots show Src phosphorylation at Tyr‐416 was indeed significantly increased when WT cells were treated with a sublethal concentration of doxorubicin (*p* < .001). On the other hand, increased Src phosphorylation at Tyr‐416 was disrupted in MMP‐2 KO cells when treated with the same doxorubicin concentration (*p* > .05) (Figure 3B).

Previous research also revealed that increases in Src phosphorylation at Tyr‐527 are observed in cases when Src is inhibited by specific upstream kinases.^27^ To determine whether Src at Tyr‐527 was phosphorylated in the WT or MMP‐2 KO cells, the levels in both untreated and 0.4 μM doxorubicin treated cells were examined (Figure 3A,B). Western blots and corresponding quantifications show non‐significant change in phosphorylation of Src at Tyr‐527 among the WT and MMP‐2 KO, either untreated or treated with doxorubicin (*p* > .05) (Figure 3A,B). Therefore, the results suggest the disruption of Src phosphorylation at Tyr‐416 in MMP‐2 KO cells is not as a result of increased phosphorylation at Tyr‐527 residue. However, our results indicate that MMP‐2 gene is an upstream mediator of a signaling pathway that regulates doxorubicin‐induced activation and phosphorylation of Src at Tyr‐416 in osteosarcoma cells.

### 3.4 MMP‐2 downregulates expression of Src family kinase inhibitors

**FIGURE 4:**
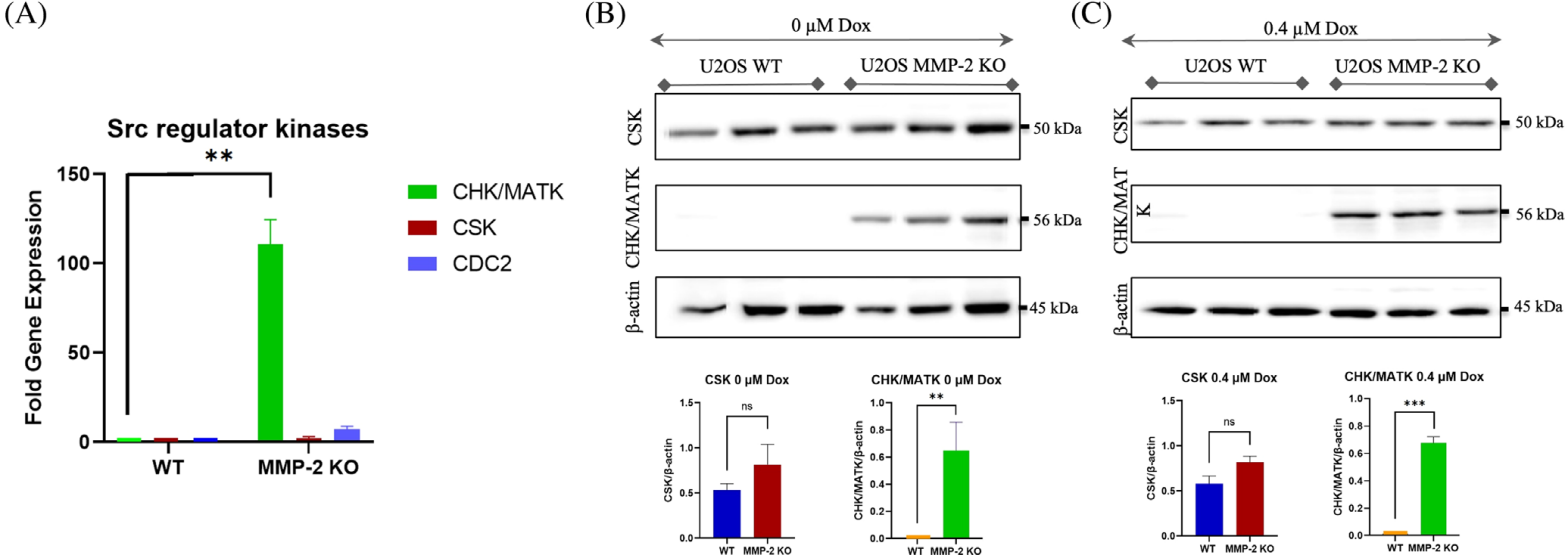
MMP‐2 knockout upregulates expression of Src Family Kinase inhibitors. (A) Fold gene expression of endogenous Src kinase inhibitors (CHK/MATK, Csk, CDC2), showing significant upregulation of CHK/MATK in U2OS MMP‐2 KO, compared to WT cells (*p* < .01, one‐way ANOVA). (B) Top; protein levels of CSK and CHK/MATK in U2OS WT and MMP‐2 KO cells. Bottom; quantification of CSK and CHK/MATK protein levels to 𝛃‐Actin levels in WT and MMP‐2 KO cells (*n* = 3). (C) Top; protein levels of CSK and CHK/MATK in 0.4 μM doxorubicin treated U2OS WT and MMP‐2 KO cells. Bottom; quantification of CSK and CHK/MATK protein levels to 𝛃‐Actin levels in 0.4 μM doxorubicin treated U2OS WT and MMP‐2 KO cells. ns *p* > .5, **p < .01, ***p < .001 (t‐test). Loss of MMP‐2 gene caused significant re‐expression of the endogenous SFK inhibitor, CHK/MATK.

To elucidate the mechanism underlying the lack of Src phosphorylation at Tyr‐416 in MMP‐2 KO cells, we analyzed the expression and activity of upstream endogenous SFK inhibitors at both the RNA and protein levels. Based on current literature, we examined three important regulators of the Src Family Kinases: CHK/MATK, Csk and CDC2.^13^, ^28^, ^29^, ^30^ The qPCR showed a significant upregulation of the CHK/MATK gene expression in the MMP‐2 KO cells compared to WT cells (110‐fold increase) (*p* < .01) (Figure 4A). Both CDC2 and Csk transcripts were also slightly upregulated in the MMP‐2 KO cells (7.0‐fold and 2.1‐fold, respectively) (*p* = .0367 and *p* = .0277, respectively). Nonetheless, our attention was drawn to the substantial increase in CHK/MATK expression within the MMP‐2 KO cells (Figure 4A).

Due to the significance of both SFK inhibitors, CSK and CHK/MATK, protein levels were measured, as shown in the western blot in Figure 4B. CSK protein levels were not significantly different between the WT and MMP‐2 KO conditions in either untreated or 0.4 μM doxorubicin‐treated cells (*p* > .05). CHK/MATK protein levels, however, were only detected in the MMP‐2 KO cells and were significantly upregulated compared to the WT cells in both doxorubicin‐treated and untreated cells (Figure 4B,C) (*p* < .01 and *p* < .001, respectively). The increased expression of CHK/MATK, a SFK inhibitor, in MMP‐2 KO cells may explain the lack of Src phosphorylation/activation by doxorubicin in these cells. This also suggests that MMP‐2 regulates the expression of CHK/MATK gene in osteosarcoma.

### 3.5 CHK/MATK overexpression inhibits osteosarcoma cell migration induced by sublethal concentration of doxorubicin

**FIGURE 5:**
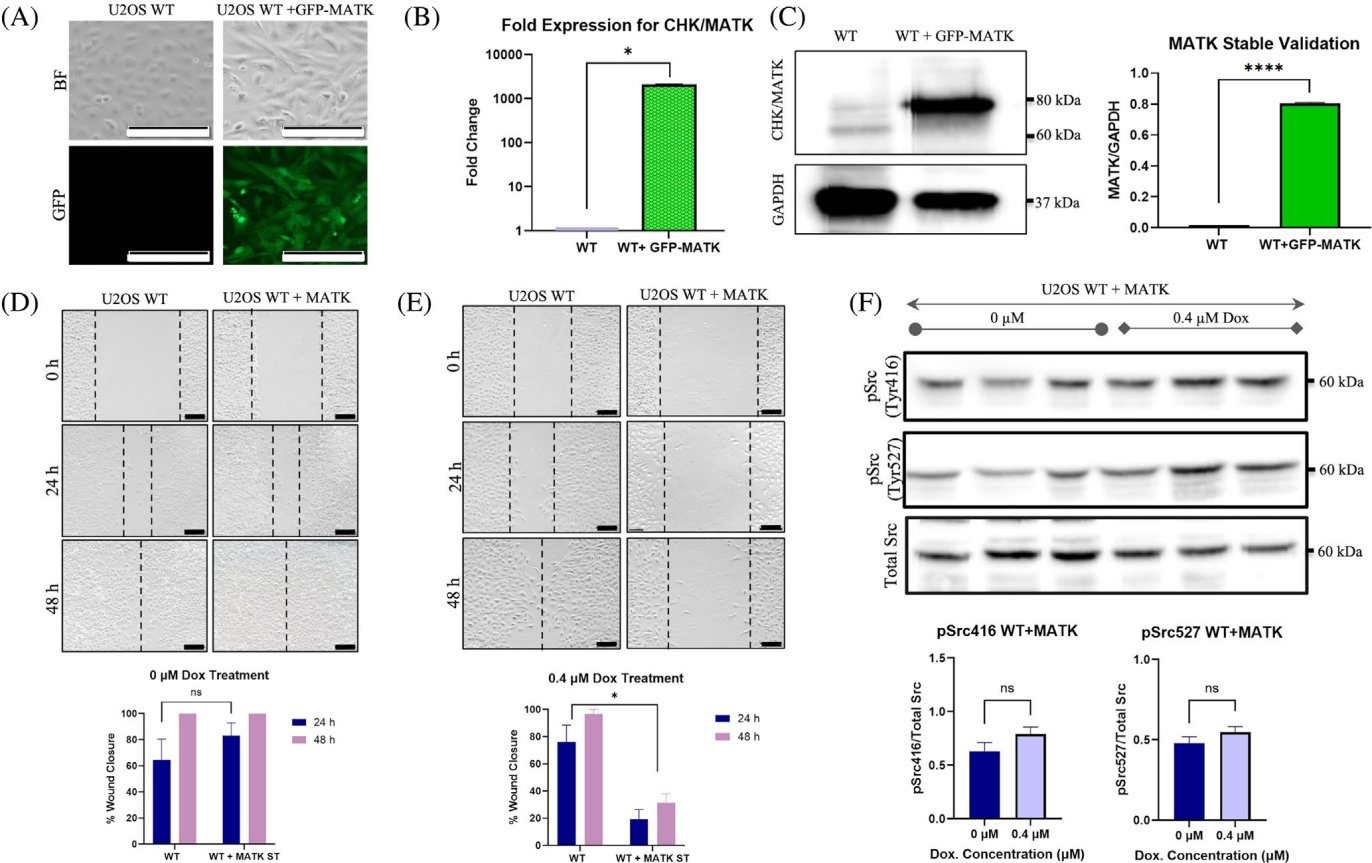
Overexpression of CHK/MATK impedes osteosarcoma cell migration in the presence of a sublethal concentration of doxorubicin. (A) GFP‐expression of U2OS WT + GFP‐MATK stables, in comparison to U2OS WT, in both GFP and bright field (White scale bar at bottom right corner = 200 μm). (B) gene fold expression for CHK/MATK in WT + GFP‐MATK compared to WT (*p* < .05, t‐test). (C) Left; protein expression for CHK/MATK in WT and WT + GFP‐MATK, validating expression in WT + MATK stables. Right; Quantification of CHK/MATK protein levels to GAPDH in WT and MATK stable cell lines (*p* < .0001, t‐test). (D) Top; wound closure assays comparing untreated U2OS WT and U2OS WT + MATK stables, showing complete closure at 48 h at 10× magnification. Black scale bar in right bottom corner of each image represents 200 μm. Bottom; quantification of % of wound closure of WT and WT + MATK stables at 24 and 48 h (*n* = 3) (*p* > .05, one‐way ANOVA). (E) Top; wound closure assays for 0.4 μM doxorubicin treated U2OS WT and U2OS WT + MATK stables at 10× magnification. Black scale bar in right bottom corner of each image represents 200 μm. Bottom; quantification of % wound closure of U2OS WT and U2OS WT + MATK stables at 24 and 48‐h time points (*p* < .05, one‐way ANOVA). (F) Top; levels of Src phosphorylation at Tyr‐416 and Tyr‐527 after 0 μM and 0.4 μM treatment at 24 h for U2OS WT + MATK Stable cells. Bottom; quantification of pSrc at Tyr‐416 (left) and pSrc at Tyr‐527 (right) for U2OS WT + MATK Stable cells for both treatments (*p* > .05, t‐test). Results show significant inhibition of cell migration in the WT + MATK stables when treated with 0.4 μM doxorubicin.

To determine the effect of overexpressing CHK/MATK in osteosarcoma, we transduced WT cells with GFP‐MATK lentiviral particles and created a stable cell line (Figure 5A). WT + MATK stable cell line was validated with qPCR and western blot, showing the significant overexpression of CHK/MATK, compared to WT (Figure 5B,C) (*p* < .05 and *p* < .0001, respectively). After validation, we conducted wound closure assays on U2OS WT and WT + MATK stable cells with and without 0.4 μM doxorubicin treatment for 48 h (Figure 5D,E). The untreated wound closure assay did not show a significant difference in cell migration between U2OS WT and WT + MATK stable cell lines as these CHK/MATK cells were able to migrate (Figure 5D) (*p* > .05). Interestingly, however, when WT + MATK cells were treated with 0.4 μM doxorubicin, we observed a significant reduction, rather than enhancement, in cell migration, with only 30% wound closure observed (Figure 5E) (*p* < .05). Our results indicate that the overexpression of CHK/MATK in U2OS cells efficiently hinders cell migration induced by sublethal concentrations of doxorubicin.

To investigate the role of CHK/MATK in inhibiting the phosphorylation of Src at Tyr‐416, we treated WT + MATK stable cells with 0.4 μM doxorubicin and examined pSrc Tyr‐416 levels. Our results indicated that doxorubicin treatment caused minimal phosphorylation at Tyr‐416, which was not significantly different from the untreated WT + MATK cells (Figure 5F) (*p* > .05). We also examined the phosphorylation level of Tyr‐527 to determine the inhibitory mechanism of CHK/MATK in osteosarcoma. Likewise, western blot analysis showed non‐significant phosphorylation at Tyr‐527 with or without doxorubicin in the presence of CHK/MATK overexpression (Figure 5F) (*p* > .05). These results suggest that the inhibitory effect of CHK/MATK on Src phosphorylation on Tyr‐416 is not due to an increase in phosphorylation at Tyr‐527. Overall, our results demonstrate that MMP‐2 knockout resulted in substantial re‐expression of CHK/MATK in U2OS cells. This re‐expression plays a crucial role in inhibiting cell migration induced by sublethal concentrations of doxorubicin by regulating Src phosphorylation/activation.

## 4 DISCUSSION

Osteosarcoma, a prevalent bone cancer in adolescents, continues to exhibit high mortality rates of 30%–40%, with metastasis occurring in at least 25% of patients at diagnosis.^30^ Although MMP‐2 is known to degrade the extracellular matrix and facilitate cancer cell invasion and metastasis, targeting extracellular MMPs clinically has proven insufficient in inhibiting metastasis.^3^ Recent findings indicate that MMP‐2 is also present in various subcellular compartments, including the nuclei of various cells such as osteosarcoma U2OS cells.^7^, ^31^ We previously reported that nuclear MMP‐2 can regulate gene expression of ribosomal RNA.^7^ Thus, examining the role of intracellular/nuclear MMP‐2 in cancer cell migration pathways is crucial for developing effective osteosarcoma treatments targeting metastasis.

We here investigated the consequences of MMP‐2 knockout on U2OS cell migration. We observed that wild‐type (WT) cells exhibited complete wound closure within 48 h, whereas MMP‐2 knockout (KO) cells showed only 35% closure. Moreover, KO cells were unable to migrate in the presence of externally supplemented active MMP‐2 within the culture media. These findings imply that the MMP‐2 gene significantly hinders osteosarcoma cell migration, with extracellular MMP‐2 having a minimal impact. We propose that the observed effect is attributed to the presence of intracellular/nuclear MMP‐2. This protease, typically involved in the degradation of extracellular matrix components, has been found to play a role in various subcellular compartments including the nucleus.^7^ The presence of intracellular/nuclear MMP‐2 in this context could potentially contribute to the enhanced cell migration observed in osteosarcoma cells treated with sublethal concentrations of doxorubicin. Further investigation is necessary to fully understand the underlying mechanisms and the role of MMP‐2 in modulating osteosarcoma cell migration. Our observations align with a previous study on retinoblastoma (RB) that investigated the effects of inactivating MMP‐2 or MMP‐9 in cell migration, invasion, and angiogenesis.^32^ These studies, collectively, indicate that targeting the MMP‐2 gene is crucial for impeding cancer cell migration.

At present, doxorubicin is a frequently utilized anticancer chemotherapy for treating osteosarcoma.^21^ Although low doxorubicin doses cause reduced cardiotoxic effects, numerous studies have reported that sublethal concentrations of doxorubicin may enhance cancer cell migration and lead to chemoresistance.^11^, ^23^ Accordingly, exploring methods to boost the efficacy of this low‐dose treatment in osteosarcoma cases is necessary. Huang et al. investigated a mechanism linked to chemoresistance, which involves the DNA‐binding protein HMGB1.^21^ As one of the three anticancer drugs, doxorubicin triggers the upregulation of HMGB1 in osteosarcoma cells. This action leads to the formation of a complex with the autophagy regulator Beclin1, thereby increasing chemoresistance. Furthermore, suppressing HMGB1 effectively restored chemosensitivity in osteosarcoma cells to lower concentrations of doxorubicin.^21^ In a related study, Tian et al. observed a similar chemoresistance effect of doxorubicin on osteosarcoma cells. They discovered that doxorubicin treatment promoted stem cell‐like characteristics in osteosarcoma, such as enhanced cell migration and proliferation, which ultimately resulted in resistance to the drug.^33^ Additionally, Tian et al. found that apatinib effectively deactivated the STAT3/Sox2 pathway and reduced doxorubicin‐induced cell migration and chemoresistance.^33^ We investigated the effects of sublethal doxorubicin concentrations on osteosarcoma cells lacking the MMP‐2 gene to identify an additional target for enhancing doxorubicin effectiveness. Our results show that MMP‐2 knockout prevents increased cell migration in response to sublethal doxorubicin concentrations. Additionally, we demonstrated that MMP‐2 gene is upstream mediator of Src kinase in the migration pathway, thus we suggest that MMP‐2 expression is not only downstream of Src activation as previously reported.^23^

We also found that MMP‐2 regulates Src kinase activity via suppression of the endogenous Src inhibitor, CHK/MATK, in osteosarcoma cells. Cheuh et al. identified that CHK/MATK is suppressed and epigenetically silenced in human colorectal cancer cells, suggesting its potential role as a tumor suppressor.^18^ To investigate MMP‐2′s role in regulating the expression of endogenous Src inhibitors Csk and CHK/MATK in osteosarcoma, we analyzed gene fold expression and protein levels in U2OS WT and MMP‐2 KO cells. While Csk gene fold expression was slightly elevated in MMP‐2 KO cells, CHK/MATK expression was dramatically increased. Protein level analysis showed a substantial upregulation of CHK/MATK in MMP‐2 KO cells, with Csk levels remaining similar between WT and KO cells. Under conditions where CHK/MATK level is elevated, achieved through MMP‐2 knockout or CHK/MATK overexpression, sublethal concentrations of doxorubicin fail to activate Src kinase activity. These findings suggest that intracellular/nuclear MMP‐2 is responsible for downregulating and suppressing the potential tumor suppressor CHK/MATK in osteosarcoma. Furthermore, the enhanced osteosarcoma cell migration induced by sublethal doxorubicin concentrations can be overcome by overexpressing CHK/MATK in WT cells. Thus, we propose that the MMP‐2 gene is an additional target to consider, as it influences the gene and protein expression of the tumor suppressor CHK/MATK in osteosarcoma. Clinically, we anticipate that inhibiting intracellular/nuclear MMP‐2 and allowing CHK/MATK re‐expression will enhance the effectiveness of sublethal doxorubicin treatments in osteosarcoma patients.

In an intriguing study by Zhu et al., it was observed that the expression of CHK/MATK is present in both normal human and mouse colon cells, where it plays a role in controlling Src activity. However, in the case of colon cancer, CHK/MATK is selectively switched off, a phenomenon that coincides with Src activation. Zhu et al. also noted a colocalization of CHK/MATK and Src in normal colon cells, where it acts to inhibit Src activity, similar to the effects witnessed in cancer cells.^34^ MMP‐2, while typically expressed in non‐cancerous cells, is found to be significantly overexpressed in many types of cancer.^6^ Uniquely, our study is the first to demonstrate that the inactivation of the MMP‐2 gene leads to the re‐emergence of CHK/MATK expression, which assists in modulating Src activity.

In addition, we analyzed the effects of overexpressing CHK/MATK in U2OS WT cells to unveil the impact on osteosarcoma migration. Surprisingly, simply overexpressing CHK/MATK in U2OS WT cells did not reduce their migration. However, CHK/MATK overexpression was able to inhibit the doxorubicin‐induced enhancement of cell migration. Previous research shows in normal untreated cells, SFKs remain in the stable inactive conformation until activated in some cellular events, including doxorubicin treatment and cell migration.^17^, ^23^ We then activated the Src system through sublethal concentrations of doxorubicin, as shown in our earlier experiments, and therefore, examined Src phosphorylation at Tyr‐416 and Tyr‐527 in our U2OS WT cells overexpressing CHK/MATK cells. In parallel with our data showing that CHK/MATK overexpression inhibited doxorubicin‐induced cell migration, there was no effect on Src activation/phosphorylation when these cells were treated with sublethal concentration of doxorubicin. Additionally, unlike Csk, CHK/MATK is also known to suppress multiple active forms of SFKs by a non‐catalytic mechanism that directly binds to the C‐terminal tail of SFKs. This inhibiting mechanism is independent of any Src phosphorylation that occurs at Tyr‐527. Despite the lack of phosphorylation at Tyr‐527, we also observed non‐significant change in phosphorylation at Tyr‐416 when cells overexpressing CHK/MATK were treated with doxorubicin. As a result, overexpressing CHK/MATK inhibits doxorubicin‐induced Src activation and phosphorylation at Tyr‐416 as well as the enhancement of cell migration in osteosarcoma.

Correspondingly, Pichot et al. investigated the effects of targeting SFKs with dasatinib in combination with doxorubicin treatments to inhibit the migration and invasion of breast cancer cells. A synergistic effect between dasatinib and doxorubicin treatments were observed, resulting in inhibiting cell migration, proliferation, thus significantly reducing IC_50_ of doxorubicin.^35^ We also report similar effects of overexpressing CHK/MATK to inhibit doxorubicin‐induced Src activation and U2OS cell migration.

## 5 CONCLUSIONS

**FIGURE 6:**
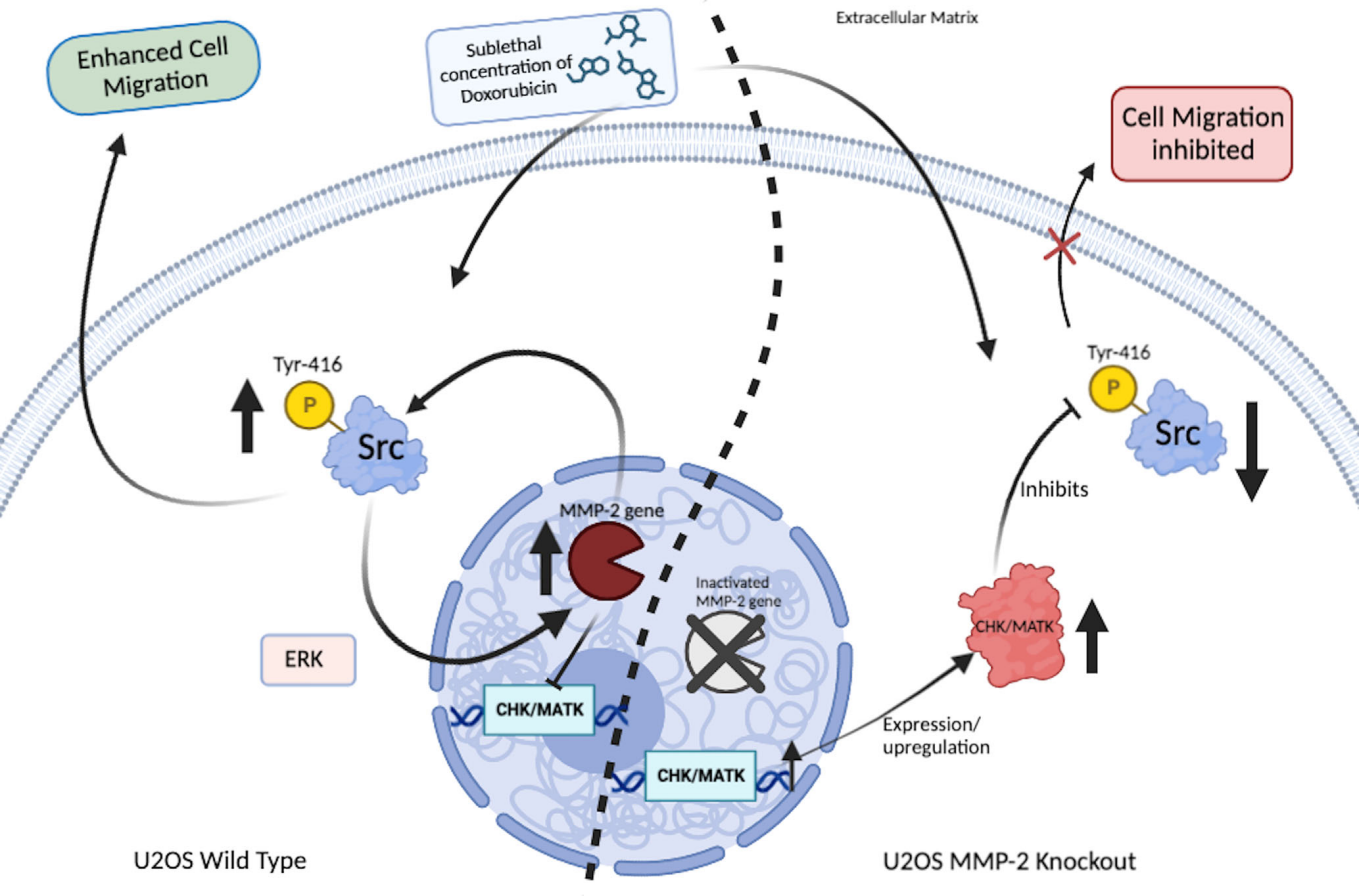
Graphical abstract. Left; MMP‐2 gene represses CHK/MATK expression. This suppression allows for activation of Src by sublethal concentrations of doxorubicin. Right; inactivation of MMP‐2 gene allows for re‐expression of CHK/MATK by its turn inhibit doxorubicin‐induced Src activation and cell migration.

This study demonstrates that the MMP‐2 gene regulates Src kinase activity by suppressing CHK/MATK, an endogenous inhibitor of Src, in osteosarcoma cells. As shown in Figure 6, MMP‐2 is upstream of Src in this pathway. Furthermore, inhibiting MMP‐2 gene expression or overexpressing CHK/MATK was able to overcome the increased cell migration caused by sublethal doses of doxorubicin. Based on these findings, we propose that targeting intracellular/nuclear MMP‐2 may be a novel therapeutic strategy, as it impacts CHK/MATK expression and activity, a known tumor suppressor in osteosarcoma. Clinically, inhibiting MMP‐2 could increase CHK/MATK levels and activity in patients. This may improve treatment outcomes for osteosarcoma when combined with lower, less toxic doses of doxorubicin. Overall, targeting the MMP‐2/CHK/MATK pathway could allow for more effective and better tolerated osteosarcoma treatment regimens compared to doxorubicin alone. Our study was conducted in cell line model of osteosarcoma, therefore, further preclinical and clinical studies are warranted to evaluate MMP‐2 inhibitors as potential adjuvants to chemotherapy in osteosarcoma.

## AUTHOR CONTRIBUTIONS

**Deanna V. Maybee:** Data curation (equal); formal analysis (equal); methodology (equal); writing – original draft (equal). **Christopher R. Cromwell:** Methodology (equal); validation (equal); writing – review and editing (equal). **Basil P. Hubbard:** Funding acquisition (equal); supervision (equal); writing – review and editing (equal). **Mohammad A. M. Ali:** Conceptualization (lead); funding acquisition (equal); project administration (lead); supervision (equal); writing – review and editing (equal).

## CONFLICT OF INTEREST STATEMENT

The authors have stated explicitly that there are no conflicts of interest in connection with this article.

## ETHICS STATEMENT

This study was approved by the Institutional Biosafety Committee (IBC) of The Binghamton University, State University of New York.

## Data Availability

Data sharing is not applicable for this article.
